# Screening, Risk Reduction Strategies, and Clinical Management of Unaffected Carriers of *BRCA1* or *BRCA2* Pathogenic Variants

**DOI:** 10.14740/wjon2692

**Published:** 2025-12-17

**Authors:** Hikmat Abdel-Razeq, Razan Mansour

**Affiliations:** a Department of Internal Medicine, King Hussein Cancer Center, Amman 11941, Jordan; b School of Medicine, University of Jordan, Amman 11942, Jordan; c Department of Hematology/Oncology, Yale School of Medicine, New Haven, CT 06510, USA

**Keywords:** *BRCA1*, *BRCA2*, Germline genetic testing, Carrier, Risk-reducing surgery, Prophylactic mastectomy, Oophorectomy, BSO

## Abstract

Breast cancer remains the most common cancer globally, with approximately 5-15% of cases linked to pathogenic variants primarily in *BRCA1* or *BRCA2*. These mutations greatly increase cancer risks, highlighting the critical need for more effective screening and prevention strategies. This review aimed to summarize existing evidence and propose a comprehensive approach to reducing cancer risk in unaffected mutation carriers. A narrative review of published literature was conducted to evaluate risk-reduction strategies, including surveillance, enhanced imaging, risk-reducing surgeries, and chemoprevention. Barriers to the uptake of these strategies and the psychological impact on carriers were also examined. Annual magnetic resonance imaging (MRI) remains the most sensitive screening tool for early breast cancer detection in high-risk individuals. Selective estrogen receptor modulators (SERMs) and aromatase inhibitors have shown potential as chemopreventive agents, but uptake remains limited due to concerns about efficacy and side effects. Risk-reducing surgeries, such as bilateral salpingo-oophorectomy (BSO) and mastectomy, significantly lower the risk of breast and ovarian cancer; however, their uptake is often hindered by emotional, cultural, and financial factors. Family communication of genetic results and support by healthcare professionals are critical to encouraging preventive actions. Effective screening and risk-reduction strategies are available for *BRCA1* and *BRCA2* carriers, yet barriers to implementation persist. Personalized counseling, enhanced accessibility, and culturally sensitive education are essential to improving the adoption of these preventive measures. Further studies are needed to explore novel chemoprevention options and interventions to address the unmet needs of carriers worldwide.

## Introduction

Breast cancer remains the most frequently diagnosed malignancy in women worldwide. In 2022, almost 2.3 million cases were diagnosed, with an estimated death toll of over 666,000 [1, 2]. In 2024, approximately 311,000 new cases of invasive breast cancer and 56,500 cases of ductal carcinoma *in situ* (DCIS) will be diagnosed among US women, and 42,250 women will die from breast cancer, highlighting the importance of effective and more accessible breast cancer screening and risk-reduction strategies [3].

Lifetime risk for breast cancer varies across countries due to differences in population structure, screening practices, and access to healthcare systems. In the United States, it is estimated that one in eight women will develop breast cancer in their lifetime [3]. Over the past decade, knowledge and experience have helped identify a subgroup of women at higher risk than others. Age, reproductive background, and a family history of breast and ovarian cancer are recognized contributors to risk. Additionally, prior breast biopsies, especially those with findings like atypical hyperplasia (AH), or lobular carcinoma *in situ* (LCIS) are established risk factors.

Risk-reducing strategies to lessen the burden of breast cancer in society are highly needed but may be difficult to implement; some of the risk factors are non-modifiable and many of the modifiable risk factors require greater efforts at both individual and society levels.

Effective breast cancer risk-reduction strategies including chemoprevention and prophylactic surgery are significantly undervalued and underutilized [4]. Studies utilizing selective estrogen receptor modulators (SERMs), including tamoxifen and raloxifene, and aromatase inhibitors (AIs) such as exemestane and anastrozole, have shown significant efficacy in lowering the risk of first breast cancer among high-risk individuals [5, 6]. However, false sense of well-being and potential harm associated with the use of these medications resulted in low uptake of these strategies among high-risk women.

Hereditary breast cancer contributes to approximately 10-15% of all breast cancers, another similar or even higher proportion of cases associated with positive family history but with no identifiable cancer-predisposing genetic variants [7].

The clinical criteria for germline genetic testing have been recently expanded by many international professional societies to probably include over 80% of all newly diagnosed breast cancer patients [8, 9]. As molecular testing and gene sequencing become more affordable and available, we believe universal testing of all breast cancer patients, as currently recommended by the American Society of Breast Surgeon, will be adopted [10-12]. This increase in patient testing may result in an increase in cascade testing of relatives and thus more unaffected “healthy” mutation carriers will be identified, mostly among younger age group. Carriers of pathogenic *BRCA2* variants have a lifetime risk of developing breast cancer ranging from 38% to 84%, while the risk of developing ovarian cancer ranges from 16.5% to 27.0% [8, 9]. Carriers of *BRCA1* mutation may have higher risk of both breast and ovarian cancers [13].

## Materials and Methods

In this review, we aimed to provide an overview of the current state of research on screening and risk reduction strategies for individuals who harbor a pathogenic *BRCA1* or *BRCA2* variant but have not been diagnosed with cancer (unaffected carriers).

### Literature search strategy

We performed a narrative/scoping review of the published literature to evaluate risk-reduction strategies, including enhanced imaging surveillance, chemoprevention, and risk-reducing surgery (RRS), and to summarize reported barriers to uptake and the psychological impact on carriers. Searches were performed in PubMed, Embase, Scopus, Google Scholar, and Cochrane Library from 1990 to 2025 using the following keywords: *BRCA1*, *BRCA2*, germline genetic testing, carrier, risk-reducing surgery, prophylactic mastectomy, oophorectomy, surveillance, and bilateral salpingo-oophorectomy (BSO). Articles were considered for inclusion if they met the following criteria: 1) published in peer-reviewed journals; 2) relevant to germline genetic testing; 3) written in English.

Data were extracted and narratively synthesized to identify major themes and gaps in the literature. This approach was chosen because our objective was to summarize and interpret heterogeneous literature rather than to provide pooled quantitative estimates.

### Data extraction and synthesis

Once the studies were identified and selected, we extracted key data, including study design, sample size, main findings, and methodologies. Data were then synthesized into common themes, allowing for a comparison across different research efforts. In some instances, meta-analyses were referenced where appropriate to quantify findings and assess trends.

### Limitations

The review is subject to certain limitations, including the possibility of publication bias, language bias (since only English-language studies were included), and the exclusion of non-peer-reviewed sources.

## Who Is at Risk?

Age at breast cancer diagnosis, ethnicity, family history, and certain pathological features of breast cancer may contribute to the risk of having a cancer-predisposing genetic alteration [14, 15]. A detailed family history should help identify relatives with history of breast and other related cancers like ovarian, pancreatic, and prostate cancers. The “closeness” of the involved family members, number involved, and how young, are important factors that need to be accurately obtained. Current American Society of Clinical Oncology (ASCO) guidelines recommend that all women with breast cancer aged 65 years or younger at time of diagnosis, regardless of their personal or family history of breast or any other cancers, should undergo germline genetic testing [16, 17]. Patients older than 65 should be offered testing if they have triple-negative (TN) disease, a personal or family history that suggests the possibility of a pathogenic variant, and if they are candidates for poly (ADP-ribose) polymerase (PARP) inhibitor therapy for early-stage or metastatic disease [18-20]. In addition, all patients of Ashkenazi Jewish ancestry, and male patients regardless of their family or personal history of cancer are candidates for germline genetic testing, too. Table 1 summarizes the updated indications for germline genetic testing [21].

**Table 1:** Recommendations for Germline Genetic Testing^a^

Age	All patients ≤ 65 years
Gender	All male patients
Ancestry	All patients with Ashkenazi Jewish Ancestry
Treatment indication	1) Systemic treatment decisions using PARP inhibitors for MBC 2) Adjuvant treatment decisions with PARP inhibitors for high-risk, HER2-negative EBC
Pathology	1) TNBC 2) Multiple primary breast cancer (synchronous or metachronous) 3) Lobular breast cancer with personal or family history of diffuse gastric cancer
Family history	Family history suggests the possibility of a pathogenic variant

^a^As per the National Comprehensive Cancer Network (NCCN) guidelines. EBC: early breast cancer; HER2: human epidermal growth factor receptor-2; MBC: metastatic breast cancer; PARP: poly (ADP ribose) polymerase; TNBC: triple-negative breast cancer.

Genetic testing recommendations are currently limited to patients diagnosed with specific cancers known for their association with hereditary predisposing genes, like breast, ovarian, and pancreatic cancers. At-risk family members should also be offered the testing following extensive counseling. The decision to pursue genetic screening often depends on the patient’s awareness and level of information regarding genetic risks. Currently, awareness and understanding of genetic testing vary, as many patients may not be fully informed about their options unless prompted by their healthcare provider. Genetic counseling sessions and consultations with oncologists, geneticists, or primary care physicians are key opportunities for patients to learn about these possibilities. However, direct-to-consumer (DTC) genetic tests are becoming accessible and affordable, often being conducted without the necessary pre- or post-test counseling, as will be further explored below.

## Family Cascade Testing

### Direct invite of family members

Discussing serious health issues within families is often complex, and this complexity increases when the condition is cancer, especially if it is inherited [22]. In non-Western societies, such conversations are further hindered as cancer diagnosis is kept secret and not openly discussed with family members [23].

We conducted a study on 169 patients including 84 (49.7%) with pathogenic/likely pathogenic (P/LP) *BRCA2* variants, 42 (24.9%) with *BRCA1*, and 43 (25.4%) with non-BRCA variants. All patients were female and were young (mean age 45 ± 9.9 years). Results were communicated with family members by the majority (n = 160, 94.7%), including 642 first-degree female relatives. However, almost half of them (n = 286, 44.5%) had taken no action. Cited reasons for not undergoing genetic testing by at-risk family members included fear of receiving positive test results (54%), cost of testing (50%), unwillingness to undergo preventive measures (34%), and social stigma (15%) [24].

A study conducted in the United States surveyed 1,103 women who had BRCA testing regarding how they shared their results with relatives; 97% indicated they had informed at least one family member. Communication rates were lower among older patients, Asian race, and when testing was performed at public hospitals versus specialized cancer centers. More importantly, 75% of BRCA-positive participants reported that at least one family member pursued genetic testing. Testing rates were lower among Asian and those with lower socioeconomic status [25].

In a smaller study, researchers found that at-risk family members responded better when called for testing by the genetic counselor (27 of 32 family members underwent cascade genetic testing) compared to only 14 of 159 (9%), when the notification and request for testing was done by the proband [26].

Several studies, including ours, had shown that patients with *BRCA1* or *BRCA2* P/LP variants were more likely to inform their family members compared to those with non-BRCA variants [27, 28]. Patients’ and healthcare professionals’ unfamiliarity with non-BRCA pathogenic variants can explain such observation. Table 2 summarizes commonly encountered barriers.

**Table 2:** Commonly Encountered Barriers for Germline Genetic Testing

Patients and at-risk relatives	1) Knowledge (relevance and importance of testing) 2) Fear of discrimination 3) Fear of cancer diagnosis 4) Cost 5) Confidentiality
Provider	Knowledge: a) Whom to test? b) Testing process; c) Result interpretation and counseling
System	1) Public awareness 2) Insurance coverage (cost of testing; access to risk-reducing interventions) 3) Communication (primary provider, counselors, surgeons, oncologist) 4) Availability of genetic counselors

### DTC

DTC germline genetic testing refers to the practice of individuals seeking genetic tests that examine inherited medical problems through companies without having healthcare professionals involved in the process. Such practice is becoming increasingly affordable and popular, especially in Western societies.

In a Canadian study known as “The Screen Project”, a total of 1,269 unaffected individuals were tested for *BRCA1* and *BRCA2*, including 87 (7.0%) males. Participants registered online and were enrolled in the study between 2017 and 2019 and were followed up for 1 year after receiving their genetic test results. Testing was conducted using a saliva sample kit, which was sent directly to the reference laboratory. Pretesting counseling for all individuals and post testing counseling for mutation-negative individuals was optional and at the individual’s discretion. Family history (first- or second-degree) of breast and/or ovarian cancer was positive in 66% of the tested individuals. In total, 30 (2.4%) had pathogenic mutation in *BRCA1* (n = 14) or *BRCA2* (n = 16). Bilateral mastectomy and/or BSO were performed by 75% of the female carriers within a year of receiving a positive result. Though genetic counseling was available at no cost, it was utilized by only 5% of the non-carriers [29].

### Population-based testing

Germline genetic testing for all women, regardless of their personal history of breast cancer, referred to as population-based testing, is complex and involves considerations of cost-effectiveness, clinical utility, and ethical implications. Current clinical guidelines recommend that testing should be offered to a subset of breast cancer patients, as it may inform treatment decisions and risk management for both patients and their close relatives. However, The American Society of Breast Surgeons recommends genetic testing for all patients with breast cancer, regardless of their risk factors [4]. However, extending these recommendations to all women, irrespective of their breast cancer status, is not universally endorsed. Recent studies from the USA [30] and Canada [31] assessing population-wide multigene testing for breast cancer prevention concluded that this strategy could be more cost-effective than testing based on family history, particularly among women aged 30 - 35 years. Despite these findings, there is no consensus that all women should undergo genetic testing. The decision to implement population-based genetic testing must consider factors such as the potential psychological impact of testing, the frequency of pathogenic variants in the general population, and the ability of the healthcare system to offer adequate genetic counseling and follow-up care, including risk-reducing strategies [9, 32]. Additionally, population-based testing would significantly increase the percentage of individuals with variants of uncertain significance (VUS), management of which remains a challenge, as these do not currently alter clinical management and require careful interpretation.

## Screening/Early Detection

### Screening for breast cancer

There is a broad consensus that surveillance and early detection in high-risk women should be based on annual contrast-enhanced breast magnetic resonance imaging (MRI), which is considered the most sensitive test for early detection of breast cancer [33]. Age to start routine annual MRI screening for BRCA1/2-positive women depends on personal and family history of breast or other cancers and age at first breast cancer diagnosis in the family [34]. Starting at age 40, MRI screening can be supplemented by mammography and optionally breast ultrasound, which can be done alternating at 6-month intervals [35]. Though several studies have demonstrated a lower rate of interval breast cancer among screened patients, no direct reduction in cancer mortality was observed utilizing high-risk MRI-based surveillance program [36]. One study that compared MRI screening versus control to screen high-risk women showed that MRI-screened women had more frequent node negative (69% vs. 44%, P = 0.001) and smaller tumors (87% vs. 52%, P < 0.001), and received less hormonal therapy (14% vs. 47%, P < 0.001) or chemotherapy (39% vs. 77%, P < 0.001) than controls. However, after a median follow-up of 9 years, overall survival was not significantly better in MRI-screened women (P = 0.064, hazard ratio (HR) 0.51, 95% confidence interval (CI), 0.24 - 1.06). Controls were not screened if they were younger than 50 years and screened with biennial mammography if they were ≥ 50 years. Women were matched according to their risk category; *BRCA1*, *BRCA2*, familial risk, and year and age of diagnosis [36].

A more recent study involved 2,488 young women (mean age at study entry 41.2 years) with a *BRCA1* (n = 2,004) or *BRCA2* (n = 484) variant. At least one screening MRI examination was performed on 1,756 (70.6%), while 732 (29.4%) women did not. With a mean follow-up of 9.2 years, 344 (13.8%) women developed breast cancer, and 35 women (1.4%) died of the disease. Among the group who entered the MRI-screening program, the age-adjusted HR for breast cancer mortality was 0.20 (95% CI: 0.10 - 0.43; P < 0.001) for women with *BRCA1* and 0.87 (95% CI: 0.10 - 17.25; P = 0.93) for women with *BRCA2* variants [37].

### Screening for ovarian cancer

It is now well established that routine screening for ovarian cancer in average-risk women utilizing transvaginal ultrasound, cancer antigen 125 (CA-125) testing, or their combination has no significant effect on ovarian cancer mortality [38], and thus is not recommended by the US Preventive Services Task Force (USPSTF) [39], which based their conclusion on the four largest studies including the Prostate, Lung, Colorectal and Ovarian screening trial (PLCO) [40, 41] and the UK Collaborative Trial of Ovarian Cancer Screening (UKCTOCS) [42].

Screening for ovarian cancer by transvaginal ultrasound and CA-125 is not reliable. In one study from Poland, 1,814 women with *BRCA1* pathogenic variants, with no prior ovarian cancers, and intact ovaries underwent at least one screening transvaginal ultrasound. Participants were followed from the date of first ultrasound until the date of preventive oophorectomy, death, or last follow-up. Among the group, 659 women had preventive oophorectomy with nine cases of incidental cancers identified at the time of surgery and three more during the follow-up, with two deaths from ovarian/fallopian cancers reported. On the other hand, 73 incident cancers were detected, and 27 deaths reported from ovarian/fallopian cancers among the remaining patients who chose to be on surveillance ultrasound. The HR for oophorectomy versus screening ultrasound was 0.23 (95% CI: 0.05 - 0.97; P = 0.05). The authors concluded that women diagnosed with ovarian cancer while participating in an ultrasound screening program have relatively poor survival, indicating that screening is not an effective substitute for preventive oophorectomy [43].

Another multi-institutional UK study (ALDO study) looked at “real-world” experience and cost-effectiveness of ovarian cancer surveillance in 875 women with pathogenic germline BRCA1/2 variants who opted to defer risk-reducing salpingo-oophorectomy (RRSO), and 767 (87.7%) women underwent at least one 4-monthly surveillance test. During 1,277 women-screen years, eight ovarian cancers were identified; two were occult at time of RRSO (both stage 1a), and six were screen-detected; three (50%) of them were early stage (≤ stage IIIa), and five (83%) had complete surgical cytoreduction. Authors concluded that surveillance for women who opt to defer RRSO in a “real-world” setting is feasible with a sensitivity of 87.5%, specificity of 99.9%, a positive predictive value of 75.0%, and a negative predictive value of 99.9% for detecting ovarian cancer [44]. Other researchers cautioned that the ALDO study results may be misinterpreted and could lead to false reassurance, emphasizing that screening high-risk women for ovarian cancer should not be viewed as a safe substitute for RRSO [45].

## RRS

Uptake and adherence to RRS recommendations is relatively low [46, 47]. Similar to genetic testing, it is critical to address barriers, particularly in resource-restricted societies where patients’ education, societal interaction, availability of high-end reconstructive surgical options and cost can be major contributing factors [48-50].

### BSO

While current guidelines suggest that *BRCA2* carriers may delay oophorectomy until age 45, most recommend earlier oophorectomy for *BRCA1* carriers [51]. Fallopian tubes are at risk of cancer and should be removed at time of oophorectomy, too [52]. In addition to decreasing the incidence of ovarian cancer, many studies have documented an added beneficial effect of risk-reducing BSO. One study showed a lower incidence of breast cancer, lower breast cancer-specific mortality, and lower ovarian cancer-specific mortality [53]. In a prospective, multicenter cohort study involving 2,482 women with *BRCA1* or *BRCA2* mutations, researchers assessed the impact of risk-reducing mastectomy or BSO on cancer outcomes. Among women without a prior history of breast cancer, those who underwent risk-reducing BSO had a lower risk of first breast cancer diagnosis compared with those who did not: 7% versus 23% in *BRCA2* carriers (HR: 0.36 (95% CI: 0.16 - 0.82)) and 14% versus 20% in *BRCA1* carriers (HR: 0.63 (95% CI: 0.41 - 0.96)). BSO was also associated with reduced breast cancer-specific mortality (2% vs. 6%; HR: 0.44 (95% CI: 0.26 - 0.76)), lower ovarian cancer-specific mortality (0.4% vs. 3%; HR: 0.21 (95% CI: 0.06 - 0.80)), and decreased all-cause mortality (3% vs. 10%; HR: 0.40 (95% CI: 0.26 - 0.61)) (Table 3) [54].

**Table 3:** Hidden Benefits of BSO

End points	BSO	No BSO	HR	95% CI
First diagnosis of breast cancer				
* BRCA1*	14%	20%	0.63	0.41 - 0.96
* BRCA2*	7%	23%	0.36	0.16 - 0.82
All-cause mortality	3%	10%	0.40	0.26 - 0.61
Breast cancer-specific mortality	2%	6%	0.44	0.26 - 0.76
Ovarian cancer-specific mortality	0.4%	3%	0.21	0.06 - 0.80

BSO: bilateral salpingo-oophorectomy; CI: confidence interval; HR: hazard ratio.

Another international, longitudinal cohort study enrolled 4,332 women with P/LP *BRCA1* or *BRCA2* variants but who had never had any cancer diagnosis. Women (mean age, 42.6 years) were followed up from age 35 to 75 years for incident cancers and deaths. Following a questionnaire follow-up, 2,932 (67.8%) underwent preventive oophorectomy at a mean (range) age of 45.4 (23.0 - 77.0) years. After a mean follow-up of 9.0 years, the age-adjusted HR for all-cause mortality associated with oophorectomy was 0.32 (95% CI: 0.24 - 0.42; P < 0.001). The age-adjusted HR was 0.28 (95% CI: 0.20 - 0.38; P < 0.001) for women with *BRCA1* and 0.43 (95% CI: 0.22 - 0.90; P = 0.03) for those with *BRCA2* variant. The estimated cumulative all-cause mortality to age 75 years for women with *BRCA1* variant who had an oophorectomy at age 35 years was 25%, compared to 62% for women who did not. On the other hand, the estimated cumulative all-cause mortality to age 75 years for women with *BRCA2* variant was 14% for those following oophorectomy compared to 28% for women who did not have an oophorectomy [55].

A recent meta-analysis of 21 studies that addressed prophylactic interventions (chemoprevention and RRS) for healthy women with *BRCA1* or *BRCA2* variants, focused on both cancer risk reduction and mortality outcomes. The meta-analysis revealed that RRS (BSO and RRM) significantly reduced cancer risk and mortality. Prophylactic oophorectomy significantly reduced hereditary breast and ovarian cancer (HBOC) risks, while the effect of prophylactic mastectomy on mortality was less conclusive [56].

### Risk-reducing mastectomy

Patients with *BRCA1* or *BRCA2*-mutated carriers are advised to consider prophylactic bilateral mastectomy (PBM) which has been shown to reduce the incidence of breast cancer by approximately 90% [57-59]. Although many earlier studies did not demonstrate an overall survival benefit [60], a recently presented study at the San Antonio Breast Cancer Symposium reported a survival advantage associated with RRM in younger women (≤ 40 years) with breast cancer who carry a BRCA1/2 P/LP variant. In this cohort, RRM was associated with a 35% reduction in the risk of death and a 42% reduction in the risk of breast cancer recurrence or development of a second primary malignancy. These improved outcomes were observed in carriers of both BRCA1 and BRCA2 variants [61]. In another study that looked at the uptake rate of mastectomies, and other RRSs, in a group of 1,020 individuals, 16% of them carried one or more high-penetrance hereditary cancer susceptibility genes including *BRCA1* and *BRCA2*. Among individuals with P/LP variants with a recommendation for RRS as per National Comprehensive Cancer Network guidelines, 34% (33/97) had mastectomy while 24% (23/94) had BSO during follow-up. Authors concluded that factors in addition to genetic test results and international recommendations may play an important role and motivate prophylactic surgery use [62].

A recently published study employing a pseudo-randomized trial design matched each woman who underwent RRM with a counterpart who did not, based on gene variant, birth year, and country. The cohort comprised 1,654 women, 827 in the RRM group and 827 in the control group. After a mean follow-up of 6.3 years, the RRM group experienced 20 breast cancers (predominantly occult) and two breast cancer-related deaths, compared to 100 incident breast cancers and seven breast cancer deaths in the control group (HR: 0.26; 95% CI: 0.05 - 1.35; P = 0.11) [63].

## Chemoprevention

### SERMs

Chemoprevention with SERMs, mostly tamoxifen, is an option offered to high-risk women to reduce the risk of breast cancer who wish not to have surgery. The evidence for tamoxifen benefit is based on clinical trials conducted among women from the general population, judged to be at higher risk for breast cancer based mostly on their age, family history, or prior breast pathology, and based mostly on assessment utilizing the Gail risk assessment model [64]. Tamoxifen, and other SERMs, have not been assessed for primary prevention in unaffected women with inherited *BRCA1* or *BRCA2* pathogenic variants.

#### Secondary prevention

In one study, 1,583 BRCA1 and 881 BRCA2 mutation carriers with unilateral breast cancer were enrolled in a tamoxifen prevention trial. Cox regression models, adjusted for multiple factors including geographic location, age at diagnosis, and prior bilateral oophorectomy, were used to estimate HRs for contralateral breast cancer (CBC) associated with tamoxifen use. Of the participants, 383 BRCA1 carriers (24%) and 454 BRCA2 carriers (52%) received tamoxifen following their initial diagnosis. Over 20,104 person-years of follow-up, 520 CBC events were recorded. The adjusted HRs were 0.38 (95% CI: 0.27 - 0.55) for BRCA1 carriers and 0.33 (95% CI: 0.22 - 0.50) for BRCA2 carriers. In a prospectively followed subgroup (4,392 person-years; 657 BRCA1 and 426 BRCA2 carriers), 100 CBC cases occurred, with adjusted HRs of 0.58 (95% CI: 0.29 - 1.13) and 0.48 (95% CI: 0.22 - 1.05) for BRCA1 and BRCA2 carriers, respectively [65].

#### Primary prevention

In one prospective study, 4,578 unaffected women with *BRCA1* or *BRCA2* variants were reached out by a questionnaire, 137 (3%) of whom reported tamoxifen use, 83 (2%) reported raloxifene use, and 12 used both drugs (0.3%). Tamoxifen or raloxifene was relatively short, with only 90 participants (44.6%) using it beyond 4 years and 61 (30.2%) using it for less than 2 years. Among the user group, a total of 202 participants were matched with 496 women, from the same cohort, who used neither drug. Information on cancer diagnosis during the follow-up period was collected by self-report and was confirmed by medical record review. After a mean follow-up of 6.8 years, breast cancer was diagnosed among 22 (10.9%) tamoxifen/raloxifene users and 71 (14.3%) among non-users (HR: 0.64; 95% CI: 0.40 - 1.03; P = 0.07) [6].

### AIs

In postmenopausal setting, estrogens are made available through the peripheral conversion of androgens utilizing the aromatase enzyme. AIs are used to minimize estrogen level in postmenopausal women. When used in the treatment of early-stage breast cancer, in the adjuvant settings, AIs resulted in 50% relative reduction in the risk of developing CBC [66]. Exemestane was used as a chemopreventive agent in the MAP.3 trial; postmenopausal (n = 4,560) women at higher risk for breast cancer based on age, Gail score, history of atypical ductal or lobular hyperplasia, LCIS or DCIS. At a median follow-up of almost 3 years, exemestane use was associated with significant reduction in invasive breast cancers in all study subgroups (HR: 0.35, 95% CI: 0.18 - 0.70) [67, 68].

Utilizing anastrozole, another AI, in 4,560 postmenopausal women at higher risk for breast cancer, the International Breast Cancer Intervention Study II (IBIS-II) reached similar conclusion [69]. Both studies were incorporated into the USPSTF’s updated meta-analysis on invasive breast cancer risk reduction, yielding a pooled risk ratio of 0.45 (95% CI: 0.26 - 0.70) [70]. Notably, both trials demonstrated a reduction in the risk of estrogen receptor (ER)-positive invasive breast cancer, but no significant effect on ER-negative disease.

In another study from MD Anderson Cancer Center (MDACC) and Baylor College, patients diagnosed with non-metastatic ER-positive breast cancer between 2004 and 2014 with known BRCA mutation status were reviewed and followed for a median time of 11.5 years from diagnosis to CBC or death. Among the 935 subjects included in the analysis, 53 had *BRCA1* while another 94 had *BRCA2*. Forty-three percent (n = 405) of patients received AI (or AI and tamoxifen) and 72% (n = 676) received tamoxifen. A total of 66 CBCs occurred; 10% (15/147) occurred in BRCA mutation carriers vs. 6.5% (51/788) in BRCA wild type. In multivariate analyses, BRCA status and AI use were significantly associated with CBC risk. AI use resulted in a significant reduction in the risk of CBC (HR: 0.44, P = 0.004), regardless of the BRCA mutation status while tamoxifen use was not associated with any beneficial effect [71].

The French LIBER Trial is studying the effect of letrozole, given at a dose of 2.5 mg daily for 5 years, in the primary prevention of breast cancer among 171 unaffected postmenopausal women aged 40 - 70 years with *BRCA1* or *BRCA2* mutation [72, 73].

### Denosumab

Recent studies indicate that women carrying pathogenic BRCA variants tend to have elevated estrogen levels, and their mammary stem cells are deficient in sex hormone-binding globulin (SHBG). These stem cells appear to be driven through the RANK/RANKL pathway, a mechanism similar to that observed in osteoclast activation [74-77]. The BRCA-P trial is an ongoing randomized study evaluating whether denosumab can reduce the risk of breast cancer in healthy BRCA1 germline mutation carriers. In this trial, women aged 25 - 55 years with BRCA1 variants, no history of breast or ovarian cancer, and no plans for prophylactic breast surgery are randomized to receive either denosumab 120 mg subcutaneously or placebo every 6 months for 5 years. The hope is that denosumab will disrupt RANKL/RANK-mediated stem cell stimulation and thereby lower breast cancer risk [77].

## Psychological Interventions

Psychological interventions can significantly improve adherence to preventive strategies and alleviate anxiety in BRCA-positive patients, who face heightened risks of several cancers including breast and ovarian cancers. Studies have shown that mindfulness-based interventions and psychological support, including cognitive-behavioral therapy, can reduce distress by helping patients process and understand their genetic information and associated risks [78]. Coping mechanisms can be significantly enhanced with these interventions, thereby enhancing emotional well-being and mitigating the psychological burden of decision-making regarding prophylactic surgeries or surveillance [79]. Additionally, social support and tailored counseling can augment motivation and thus improve adherence to preventive strategies such as surveillance imaging, RRSs, or chemoprevention [80]. This holistic approach may better optimize long-term health outcomes in BRCA-positive unaffected carriers and even patients with cancer.

## Cultural Issues

The implementation of risk-reduction strategies in patients with *BRCA1* or *BRCA2* mutations may be significantly influenced by cultural factors. Cultural beliefs about health, family, and body image can shape how patients perceive their risks and the acceptability of surgical interventions while healthy and before they develop cancer. In some cultures, there may be strong emphasis on fertility and motherhood, leading to reluctance in opting for procedures like prophylactic oophorectomy or mastectomy [81]. Additionally, cultural differences in understanding preventive measures to manage cancer risks may lead to disparities in access to genetic testing and surgery [82]. Studies have shown that fear of disfigurement, cultural stigma around cancer, and religious or familial pressures may prevent women from pursuing risk-reduction surgeries [83]. These cultural barriers underscore the need for healthcare providers to adopt culturally sensitive approaches when counseling patients about BRCA-related risk-reducing options, ensuring that societal influences and personal values are considered in the decision-making process [84].

## VUS

VUS are genetic changes identified through genetic testing that cannot currently be classified as benign or pathogenic. A VUS may be found in a gene known to be associated with a disease, but there is insufficient evidence to confirm whether the specific variant causes or contributes to the disease. The classification of VUS is a crucial part of genetic testing and interpretation. The criteria for VUS classification are usually based on guidelines set by professional bodies and usually depend on the frequency of the detected variant in the general population in public database like the ClinVar, variant’s location within the gene, functional studies, family segregation studies, and scientific literature reports. As new data, research, and technological advancements become available, VUS classifications may change. Re-evaluating VUS helps improve the accuracy of clinical decision making, counseling, and further genetic testing [85]. The management of VUS in clinical practice can be challenging due to the uncertainty about their clinical impact. However, several specific strategies can help guide clinical decisions and ensure the best possible care for patients. These strategies involve careful interpretation, follow-up, patient and family education, psychosocial support, and coordination with various healthcare professionals. It is important to keep patients informed about the reclassification status of their variants and they should be empowered to make decisions about their healthcare based on the most current information. Clinicians should document the VUS and any follow-up actions clearly, including any changes in the interpretation over time. Reporting to international registries or databases, like the ClinVar, should help collect broader population-based data on the variant [86].

## Conclusions and Future Directions

Germline genetic testing is increasingly utilized for patients with breast and many other cancers. The indications for testing were recently widened to cover most newly diagnosed patients with breast cancer and it is anticipated that universal testing of all newly diagnosed patients will soon be the standard. As we expand in cascade family screening, more “cancer-free” individuals will be identified as “carriers”. Given the high penetrance rate of both *BRCA1* and *BRCA2* variants, risk-reduction strategies for this group of unaffected participants are highly needed. Though alternatives to risk-reducing mastectomy have “reasonable” safety, RRSO might be a must after proper counseling, timing, and careful evaluation of participants’ needs. It is hoped that ongoing and future research on chemoprevention will help ease at least some of the growing concerns of patients and their at-risk family members. New molecular technologies, like liquid biopsies, may be useful in future research to help detect breast, ovarian, and other cancers at a much earlier stage among carriers of cancer-predisposing genes. More prospective, world-wide inclusive studies, addressing the same issue in resource-restricted settings are highly needed. Lastly, there is a need for standardized practices of genetic testing to ensure personalized and effective counseling and RRSs [87].

## Data Availability

The authors declare that data supporting the findings of this study are available within the article.
